# Supplementary material for: NMR Spectroscopy Identifies Chemicals in Cigarette Smoke Condensate That Impair Skeletal Muscle Mitochondrial Function
Source: Toxics. 2022 Mar 14;10(3):140. doi: 10.3390/toxics10030140 (PMC8955362; doi:10.3390/toxics10030140)
Supplement: Supplementary file 1 [file toxics-10-00140-s001.zip › toxics-1602276-supplementary.pdf]

toxics

Supplementary Materials: NMR Spectroscopy Identifies Chemicals in Cigarette Smoke Condensate That Impair Skeletal Muscle Mitochondrial Function

Ram B. Khattri [1], Trace Thome [1], Liam F. Fitzgerald [2], Stephanie E. Wohlgemuth [3], Russell T. Hepple [2] and Terence E. Ryan [1,4,*]

Table S1. Peak assignments of chemicals present in cigarette smoke condensate (CSC). Chenomx NMR Suite 8.6, biological magnetic resonance bank (BMRB) and several published literatures [1–4] were used to annotate these chemicals. The concentrations of the majority of these chemicals were determined using Chenomx NMR Suite 8.6 or by using a peak area of either 0.5 mM DSS peak at 0.0 ppm (for water soluble chemicals) or 10 mM pyrazine peak at 8.61 ppm (for chloroform soluble chemicals).

S.No.	Chemicals	"δ" in ppm & multiplicity[a]	Concentration (mM)	Solvent system
1	Glycerol [1,2]	3.8(m), 3.6(dd), 3.6(dd)	20.51	X*
2	Quinoline [3]	8.5(dd), 7.9(dd), 7.5(q)	9.22	Y*
3	Octanoic acid and/or nonanoic acid and/or decanoic acid and/or pentadecanoic acid and/or palmitic acid (Mixtures) [2]	5.08(m); 2.33(t), 1.63(p), 1.30(m), 0.87(t)	9.0	Z*
4	Acetonitrile [2]	2.05(s)	7.88	Y*
5	Acetone [3]	2.3(s)	4.68	Y*
6	Ethylene glycol	3.6(s)	2.12	X*
7	Methanol	3.22(s)	1.62	Y*
8	Butanone [3]	2.6(q), 2.2(s), 1.0(t)	1.46	Y*
9	Triacetin	5.31(m), 4.35(dd), 4.30(dd); other peaks are obscured	1.33	X, Z*
10	Pyrogallol	6.86(t), 6.76(d)	1.07	Z*
11	Lactate	1.32(d), 4.11(q)	0.77	X*
12	Phenol [3]	7.2(t), 6.9(t), 6.8(d)	0.73	Y*
13	Proline	4.1(q), 3.4(m), 3.3(m); other peaks are obscured	0.70	X*
14	Acetamide [2,3]	2.0(s)	0.69	Y*
15	Myo-Inositol	4.1(t), 3.6(t), 3.5(dd), 3.3(t)	0.68	X*
16	Nicotine [2,3]	8.66(d), 8.65(d), 8.04(d), 7.59(q); other peaks are obscured	0.65	X*
17	1,2,3,4-tetrahydronaphthalene [2]	7.15 (m), 2.76(m), 1.83(m)	0.64	Z*
18	N-Nitrosodimethylamine	3.8(s), 3.1(s)	0.59	X*
19	1,6-Anhydro-β-D-glucose	5.4(t), 4.6(d); other peaks are obscured	0.47	X*
20	Hydroquinone [2,3]	6.8(s)	0.43	X*
21	Dimethylamine	2.74(s)	0.42	X*
22	Isopropanol	3.8(m), 1.1(d)	0.4	Y*
23	Catechol [2,3]	6.9(q), 6.9 (q)	0.37	X*
24	α-pinene [2]	3.04(d), 2.02(m), 1.90(m), 1.71(s), 1.62(d), 1.33(d), 0.91(s)	0.34	Z*
25	Succinate	2.4(s)	0.31	X*
26	Citrate	2.7(d), 2.5(d)	0.30	X*
27	Ascorbate	4.5(d); other peaks are obscured	0.28	X*
28	Valerate	0.9(t); other peaks are obscured	0.27	X*
29	Butyrate	0.9(t); other peaks obscured	0.22	Y*

30	Leucine	0.9(t); Other peaks obscured	0.19	*X**
31	Cotinine [2]	8.52(d), 8.51(d), 8.46(d), 7.79(t), 7.77(t), 7.51(q), 2.61(m), 1.96(m)	0.183	*X**
32	Dimethyl sulfide	3.1(s)	0.17	*X**
33	Isoleucine	1.0(d); other peaks obscured	0.17	*X**
34	Propylene glycol [1]	3.9(m), 3.5(dd), 3.4(dd), 1.1 (d)	0.14	*X**
35	Alanine	1.46(d),3.8(q)	0.14	*X**
36	Acetate	1.91(s)	0.13	*X**
37	Valine	1.10(d), 1.0(d); other peaks obscured	0.13	*X**
38	Isovalerate	0.9(d); other peaks obscured	0.11	*X**
39	o-cresol [3]	7.2(d), 7.1(t), 6.9(d), 6.9(d), 2.2(s)	0.087	*X**
40	2-Furoate	7.6(s), 7.0(d), 6.5(q)	0.08	*X**
41	Ethyl benzene [3]	1.00(t), 2.8(q), 7.1(s)	0.08	*Z**
42	N-nitrosomorpholine [2]	4.31(t), 3.90(t), 3.87(t), 3.67(t)	0.07	*Z**
43	p-cresol [2,3]	7.1(d), 6.8(d), 2.2(s)	0.06	*X**
44	Sucrose	5.4(d); other peaks obscured	0.06	*X**
45	Formaldehyde [2,3]	9.6(s)	0.06	*Z**
46	Glucose	5.2(d); other peaks obscured	0.06	*X**
47	Tyrosine	7.2(d), 6.9(d), 3.9(q), 3.2(q), 3.0(q)	0.04	*X**
48	Pyridine [2,3]	7.45(t), 7.90(m), 8.45(d)	0.04	*X**
49	Phenylacetate [2]	7.4(t), 7.3(t), 3.5(s)	0.04	*X**
50	4-Hydroxybenzoate	6.9(d), 7.8(d)	0.03	*X**
51	Nicotinate	8.9(dd), 8.6(dd), 8.2(dt), 7.5(dd)	0.02	*X**
52	Formate	8.45(s)	0.02	*Y**
53	Niacinamide	8.9(d); 8.2(dt); other peaks are obscured	0.02	*X**
54	Cis-Aconitate	5.7(t); 3.1(d)	0.01	*X**
55	Caffeine [2,3]	7.9(s); other peaks obscured	0.01	*X**
56	1-Methylnicotinamide	9.3(s), 9.0(d), 8.9(d); other peaks obscured	0.007	*X**
57	Benzaldehyde [4]	10.05(s); other peaks are obscured	0.006	*Z**
58	Propionaldehyde [3]	1.02(t), 2.47(q), 9.74(s)	0.005	*Z**
59	Nonanal [2]	9.76(s); other peaks are obscured	0.003	*Z**
60	Octanal [2]	9.77(s); other peaks are obscured	0.002	*Z**

[a]Multiplicity of 1D ^{1}H resonances: "s", singlet; ''d", doublet; "t", triplet; "dd", doublet of doublet; "q", quartet; "m", multiplet. Solvent system: "X*" represents the NMR sample that was prepared after flash freeze drying of 45 μL of 4% CSC followed by re-suspension in 50 μL of 50 mM phosphate buffer (pH 7.2) along with 0.5 mM of DSS and 0.02% NaN_3 in 100% deuterated condition. "Y*" consisted of 50 μL of NMR sample prepared by taking 45 μL of 4% CSC combined with 0.5 mM of DSS and 0.02% NaN_3. A small drift in the chemical shift of some chemicals can be seen because of the acidic pH of this solvent system. "Z*" solvent system had NMR sample that was prepared by flash freeze drying of 45 μL of 4% CSC followed by resuspension in 70 μL of $CDCl_3$ with 10 mM of pyrazine as an internal standard. Note: Triacetin was found in both aqeuous and organic phases.

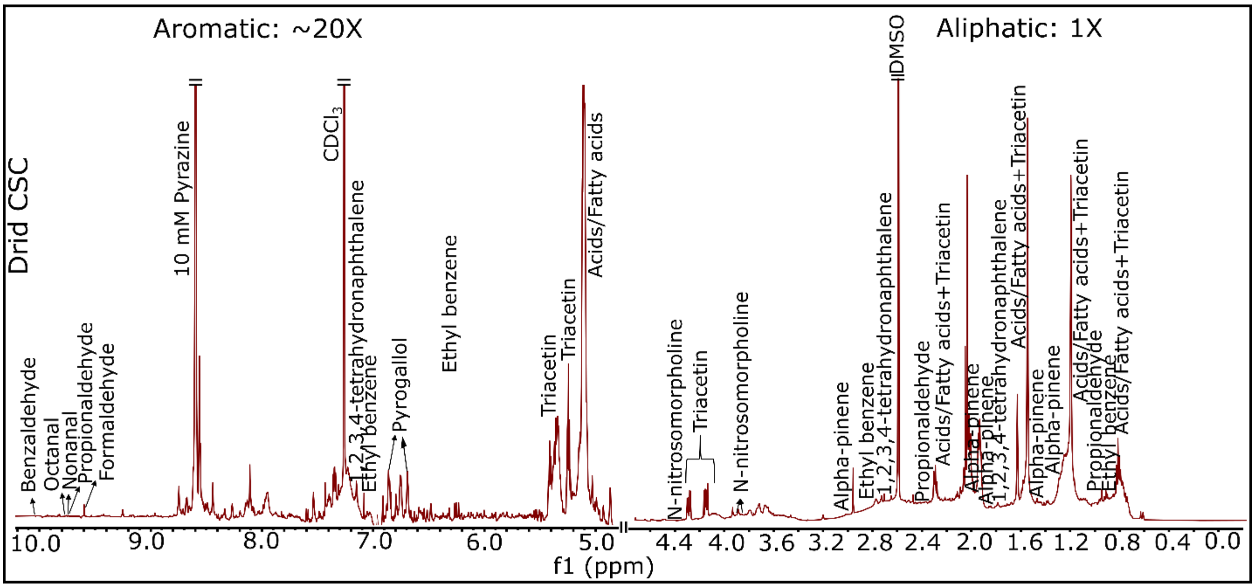

Figure S1. ^{1}H NMR spectra showing the hydrophobic metabolites (in $CDCl_3$ with 10 mM of pyrazine as an internal standard) in 4% cigarette smoke condensate. A total of 12 metabolites were annotated (listed in Supplementary Table S1) using Chenomx NMR Suite 8.6, biological magnetic resonance bank (BMRB), a set of 2D experiments (Supplementary Figures S2-S4), and several published literatures.

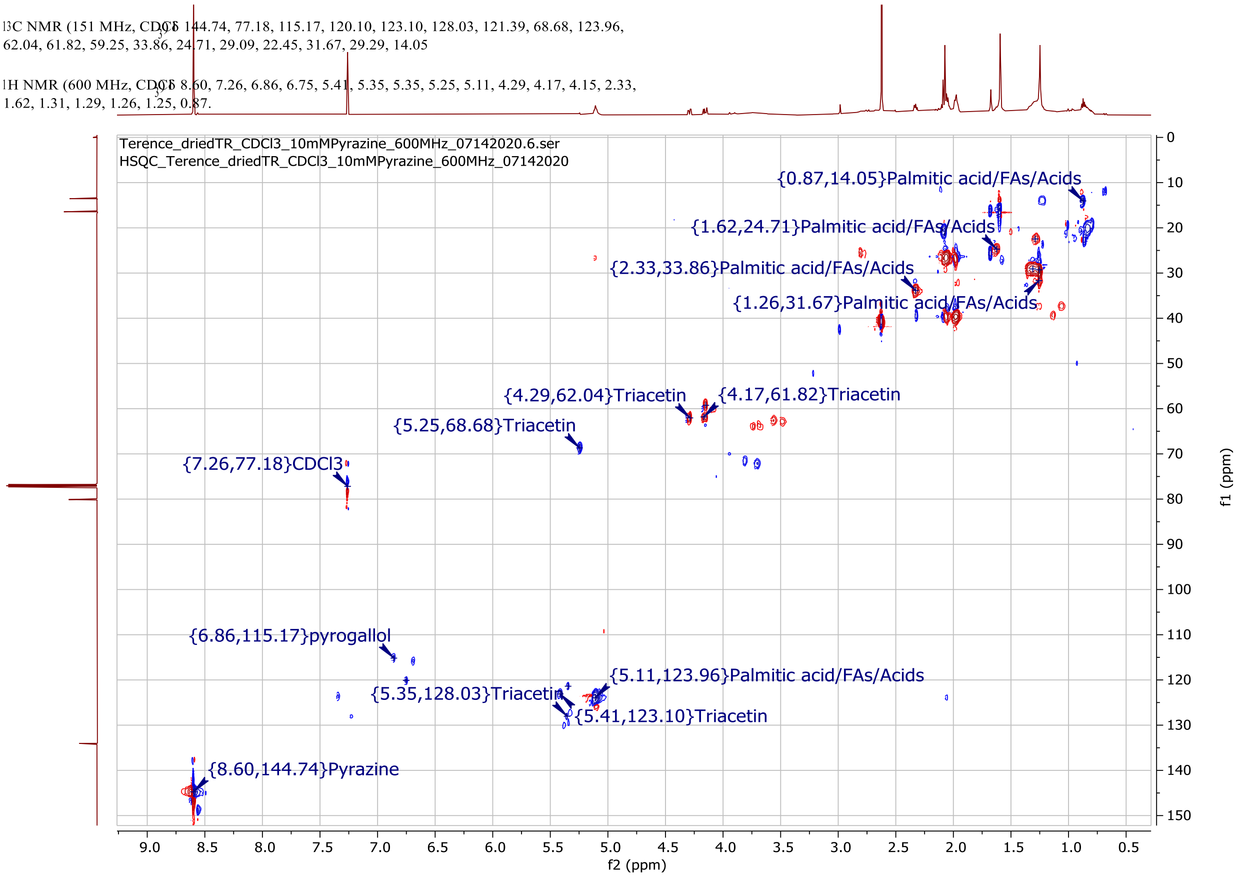

Figure S2. A part of heteronuclear single quantum coherence spectroscopy (HSQC) showing hydrophobic metabolites acquired with a CP TXI CryoProbe with an Avance II Console (14.1 T, Bruker Biospin, Billerca, MA) NMR instrument.

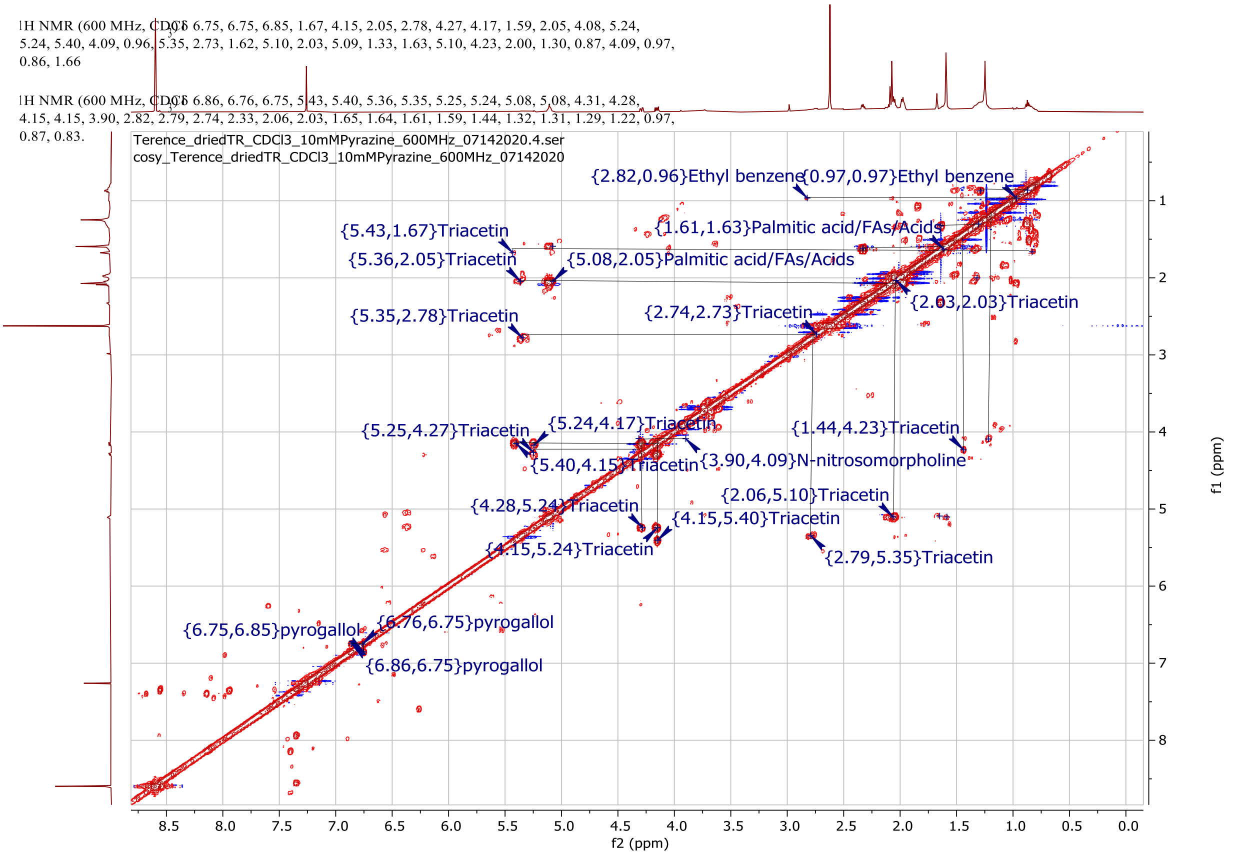

Figure S3. A part of homonuclear correlation spectroscopy (COSY) showing hydrophobic metabolites acquired with a CP TXI CryoProbe with an Avance II Console (14.1 T, Bruker Biospin, Billerca, MA) NMR instrument.

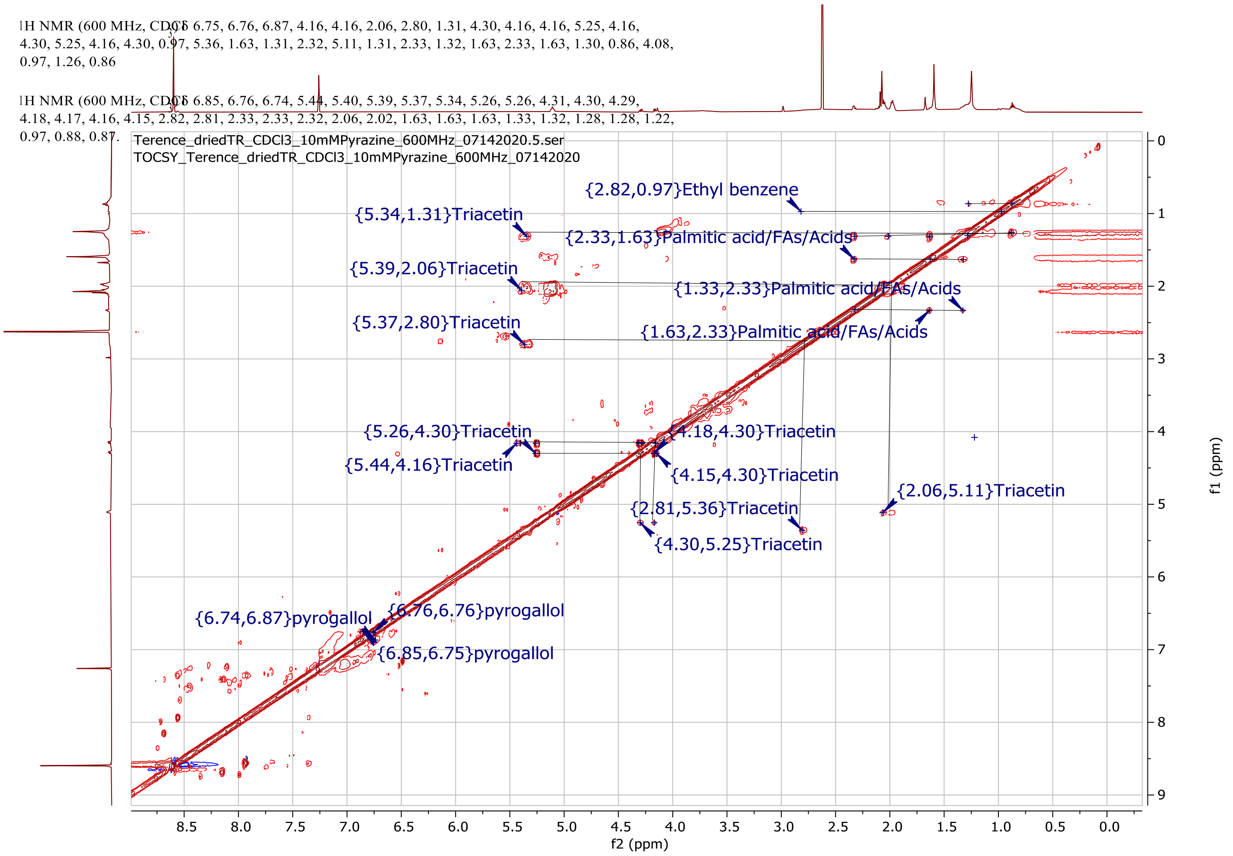

Figure S4. A part of total correlation spectroscopy (TOCSY) showing hydrophobic metabolites acquired with a CP TXI CryoProbe with an Avance II Console (14.1 T, Bruker Biospin, Billerca, MA) NMR instrument.

References

1. Jensen, R.P.; Strongin, R.M.; Peyton, D.H. Solvent Chemistry in the Electronic Cigarette Reaction Vessel. *Sci. Rep.* **2017**, *7*, 42549. https://doi.org/10.1038/srep42549
2. Ulrich, E.L.; Akutsu, H.; Doreleijers, J.F.; Harano, Y.; Ioannidis, Y.E.; Lin, J.; Livny, M.; Mading, S.; Maziuk, D.; Miller, Z.; et al. BioMagResBank. *Nucleic Acids Res.* **2008**, *36*, D402–D408. https://doi.org/10.1093/nar/gkm957.
3. Ticha, J.; Wright, C. Rapid detection of toxic compounds in tobacco smoke condensates using high-resolution H-1-nuclear magnetic resonance spectroscopy. *Anal. Methods* **2016**, *8*, 6388–6397. https://doi.org/10.1039/c6ay00849f.
4. Remaud, G.; Debon, A.; Martin, Y.; Martin, G.; Martin, G. Authentication of bitter almond oil and cinnamon oil: Application of the SNIF-NMR method to benzaldehyde. *J. Agric. Food Chem.* **1997**, *45*, 4042–4048. https://doi.org/10.1021/jf970143d
